# Does Exercising with the Use of Virtual Reality during Haemodialysis Have an Impact on Plasma Levels of Interleukin 1β, Interleukin 6, and Interleukin 8?

**DOI:** 10.3390/jcm12165358

**Published:** 2023-08-17

**Authors:** Agnieszka Turoń-Skrzypińska, Iwona Rotter, Jarosław Przybyciński, Aleksandra Szylińska, Alicja Mińko, Kazimierz Ciechanowski, Grażyna Dutkiewicz

**Affiliations:** 1 Department and Unit of Medical Rehabilitation and Clinical Physiotherapy, Pomeranian Medical University, 71-210 Szczecin, Poland; agi.skrzypinska@gmail.com (A.T.-S.); iwrot@wp.pl (I.R.); aleksandra.szylinska@gmail.com (A.S.); 2 Department of Nephrology, Transplantology and Internal Medicine, Pomeranian Medical University, 70-111 Szczecin, Poland; jaroslaw.przybycinski@pum.edu.pl (J.P.); kazimierz.ciechanowski@pum.edu.pl (K.C.); grazyna.dutkiewicz@pum.edu.pl (G.D.)

**Keywords:** dialysis, virtual reality, cytokine

## Abstract

Cytokines are a group of fine proteins which play a key role in the regulation of various biological processes, including inflammatory reactions. Proinflammatory cytokines, such as interleukin 1β (IL-1β), interleukin 6 (IL-6), and interleukin 8 (IL-8), are produced in response to various stimuli, including infections, tissue damage, and oxidative stress. Virtual reality (VR) use during intradialytic exercises improves physical activity. The purpose of the study was to evaluate the relationship between exercising regularly with the use of virtual reality during haemodialysis and the levels of selected cytokines (Il-1, Il-6, Il-8). The study and the control groups consisted of end-stage renal disease patients who underwent haemodialysis as a renal replacement treatment. The study group comprised patients subject to haemodialysis as a renal replacement therapy who were to work out with the use of a prototype of the NefroVR system for 20 min when undergoing haemodialysis (HD). Statistical analyses utilised Statistica 13. The conducted research demonstrated that regular exercises with the use of virtual reality might be related to a decrease in inflammation in patients included in the chronic haemodialysis programme. It is key to encourage patients with end-stage renal disease treated with haemodialysis to exercise regularly because of the possibility of their proinflammatory parameters becoming reduced.

## 1. Introduction

According to the definition developed by the Kidney Disease Improving Global Outcomes (KDIGOs), chronic kidney disease (CKD) is a condition which involves kidney structure or function abnormalities which persist for a period longer than three months. CKD is a significant global health issue. It is estimated that it affects circa 13.4% (between 11.7% and 15.1%) of the population worldwide [1]. Cytokines are a group of fine proteins which play a key role in the regulation of various biological processes, including inflammatory reactions. Amongst over one hundred and fifty known cytokines, there are some which influence immunological and inflammatory processes. Proinflammatory cytokines, such as interleukin 1β (IL-1β), interleukin 6 (IL-6), and interleukin 8 (IL-8), are produced in response to various stimuli, including infections, tissue damage, and oxidative stress. Their main function is the activation and homing of immune cells to infection or damage sites, which lead to inflammatory processes [2,3]. Interleukin 1 (IL-1) is the major proinflammatory cytokine which plays an important role in both acute and chronic inflammatory responses. IL-1β may induce the secretion of other inflammatory cytokines, such as IL-6. Both the former and the latter stimulate the processes of nerve tissue fibrosis. IL-1β belongs to the family of cytokines IL-1 [3,4,5] and affects the activity of the cells of the renal stroma by inducing the programme of MYC transcription-factor-dependent genes, which contribute to renal fibrosis [3]. During inflammation, the levels of various cytokines rise, including those of the pleiotropic IL-6, with its gene mapped to chromosome 7p15-p21 [6,7,8]. Already in the early stages of CKD, IL-6 increases, even more so in haemodialysis patients [9,10]. Proinflammatory factors, including IL-1β, affect the levels of IL-8, with its gene mapped to chromosome 4q12-q13 [11]. IL-8 is produced locally in the muscles during exercise, whereas a minimum systemic reaction is observed, amongst others, following intense exercise with an eccentric component due to a related proinflammatory response [12]. Typically, CKD patients demonstrate increased levels of inflammatory markers. Inflammation may be caused by several factors, such as underlying disease, circulatory diseases, concomitant diseases, complications of haemodialysis treatment, and infections. In the case of CKD patients, inflammation is oftentimes related to progressing kidney damage as well as its complications, such as arteriosclerosis and cardiovascular diseases. Increased levels of proinflammatory cytokines, e.g., IL-1β, IL-6, and IL-8, are usually associated with the enhancement of inflammation in CKD patients. Considerable enhancement of inflammation may contribute to major complications in CKD patients and may increase the risk of death. It is crucial to monitor inflammation in CKD patients and implement the right treatment to reduce the risk of developing complications and improve treatment outcomes [2,13].

In 1983, Cannon and Kluger conducted the first study which suggested that there is a relationship between physical exercise and cytokine response [14]. Physical activity has an impact on the levels of inflammatory markers both under normal and abnormal conditions. Studies demonstrate that regular physical activity can lower the concentration of proinflammatory cytokines, such as IL-1β, IL-6, or IL-8, which contributes to the reduction in inflammation in the body. Furthermore, physical activity may attenuate inflammation. In the case of chronically ill patients, for instance CKD patients, physical activity might have a beneficial effect on inflammation and can improve treatment results. Nonetheless, excessive work out may lead to exhaustion and increased oxidative stress, which in turn may augment inflammation. It is vital to choose physical activity levels suitable for a given patient, taking into consideration one’s individual needs and abilities [15].

Virtual reality (VR) is an interactive computer-generated experience based on the visual observation of objects, events, and tasks with the use of a three-dimensional effect [16,17,18,19]. Exercising while undergoing haemodialysis is beneficial: the time is used well, it is attractive for patients, and it promotes a healthy lifestyle and safety. VR use during intradialytic exercises improves physical activity, fitness, and life quality. The effects of VR systems as supplementation of rehabilitation comprise user interaction with simulated environments and real-time feedback. Thus, functional actions, which are repeatable, are ensured [19,20,21,22,23,24]. Work out with the application of virtual reality during haemodialysis means making use of the time the patient spends at a dialysis station, reducing the monotony of the dialysis therapy and its safety (medical oversight of the medical staff and accessibility of medical equipment), gaining the specified benefits of regular physical activity at an earlier point. Benefits arising from the application of virtual-reality-based exercises to improve physical fitness in dialysis patients have been confirmed [25,26].

The purpose of the study was to evaluate the relationship between exercising regularly with the use of virtual reality during haemodialysis and the levels of selected cytokines (Il-1, Il-6, Il-8).

## 2. Materials and Methods

One hundred and two (102) patients with stage 5 chronic kidney disease who underwent haemodialysis as a renal replacement therapy participated in the study conducted between March 2021 and February 2022. The study subjects were qualified for the study by a nephrologist.

**Figure 1:**
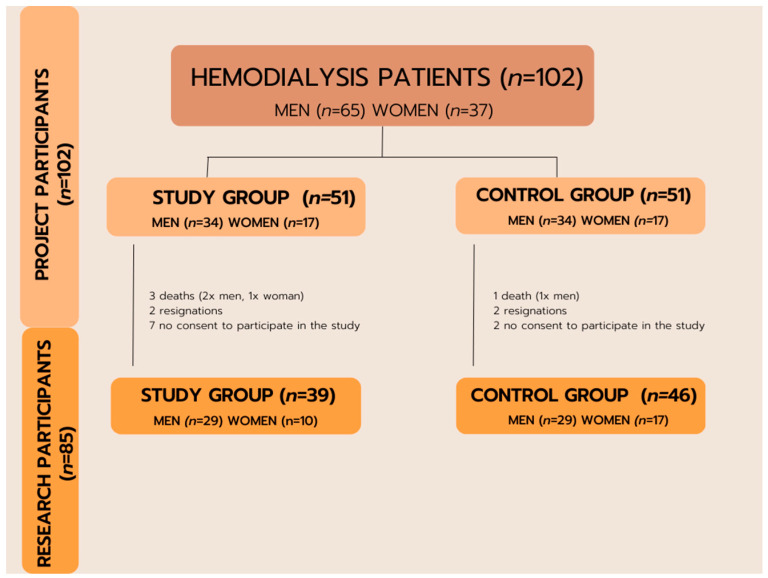
Qualification scheme for the research.

Ultimately, a total of 85 patients, 58 males and 27 females, who underwent haemodialysis as a renal replacement therapy participated in the study. The study group comprised 39 people, 29 males and 10 females. The control group comprised 46 people, 29 males and 17 females (Figure 1).

The study and the control groups consisted of end-stage renal disease patients who underwent haemodialysis as a renal replacement treatment due to the complete absence of diuresis recruited from the Nephrology, Transplantology and Internal Medicine Clinic at Pomeranian Medical University. Both groups received three haemodialysis sessions per week. The average duration of one haemodialysis session was 223.85 ± 20.47 min (mean ± SD) in the study group and 216.52 ± 28.92 min (mean ± SD) in the control group.

The group profile is presented in Table 1. Neither the study nor the control group showed statistically significant differences in the evaluation of demographic data or the evaluation of concomitant diseases.

The study group was selected based on the criteria qualifying and disqualifying participants for and from the study. The criteria qualifying individuals to participate in the research project included: written consent to participate in the study, a complete absence of diuresis, undergoing haemodialysis as a renal replacement therapy for at least 3 months (three sessions per week), and age over 18 years old. The criteria disqualifying patients from the research project comprised: no written consent to participate in the study, locomotory organ disease preventing participation in the study, serious cardiovascular diseases (NYHA III or IV cardiac insufficiency), acute coronary syndrome over the last three months, uncontrolled arterial hypertension, uncorrectable vision impairment, poorly controlled diabetes (HbA1c above 8% for 3 months), old-age dementia, other neurological or mental disorders which prevent the ability to provide a consent to examination or to understand the nature of examination and conditions of participation, malignant tumours, surgeries over the last month, or amputation of a lower limb which prevents examination or epilepsy.

### 2.1. Study Process

The studied patients were randomly divided into two groups: the study group and the control group, depending on the assigned intervention. The study group comprised patients subject to haemodialysis as a renal replacement therapy who were to work out with the use of a prototype of the NefroVR system (Szczecin, Poland) for 20 min when undergoing HD. Work outs were performed three times per week for the first (1–2) hours of HD treatment or until an ultrafiltration (UF) of 2.5 was achieved.

The control group comprised patients subject to haemodialysis as a renal replacement therapy who were not assigned an intervention.

The tests involved the use of a prototype of the NefroVR system composed of the following components placed on a mobile platform with a ballast:

- A base unit (responsible for connecting all components and initiating dedicated software);
- A rehabilitation rotor with a flywheel and manually set load (to perform exercises during dialysis);
- A VR goggles set (for the patient to experience virtual reality);
- A panoramic screen for the patient;
- A control touchscreen (for the operator—a doctor, a nurse, or a physiotherapist);
- A control panel (for the patient), digital joystick, and one button.

The device worked as an audio–visual stimulation provoking patient’s physical activity. A patient connected to the device participated in a virtual experience, in a game, where the only possibility of movement was to use the rehabilitation rotor that was formed as a part of the device. The speed of the rotor’s revolutions affects the patient’s pace in the game, whereas the doctor/physiotherapist attending to the patient convert the number of rotor’s revolution into suitable speed in the game and the physical resistance of the flywheel. The parameters had to be adjusted to patient’s current health condition so that it would not be excessive (e.g., too high a heart rate or a decrease/increase in BP below/over allowed levels). In the clinical tests phase, the patients could choose from a selection of 5 mini-games to be played for 15–20 min. In addition to controlling the game with the rotor, the patient could use extra options of interaction with a joystick and one button. The interactivity level available to a given patient was limited at the stage of clinical research to the level needed to maximally reduce the time needed to introduce the patient to Nefro VR operations.

The Bioethics Committee approval number KB-0012/144/2020 of 5.10.2020 was obtained to conduct the research study. Data were collected twice: at the time of entering the research project (E0) and following month three (E3). At the time of entering the study, the patients had to complete an author’s survey made for the needs of the study and comprising questions about participant’s demographics, health condition, and lifestyle.

The participants in the research project had selected blood biochemical parameters checked (interleukin 1β, interleukin 6, and interleukin 8 levels).

The study group and the control group subjects each had 2 mL blood specimen collected. The patient with end-stage renal disease treated with haemodialysis had their blood collected from arteriovenous dialysis shunt in the dialysis station at the Clinic of Nephrology, Transplantology and Internal Medicine, Pomeranian Medical University, into EDTA medium on the day of cyclical blood check, at E0 and E3. The collected blood samples were centrifuged (4000×*g* rpm for 10 min) at a temperature of 4 °C in the MPW—350R centrifuge (MPW MED. INSTRUMENTS, Warsaw, Poland). Blood plasma was divided into two separate Eppendorf tubes, 1.0 mL plasma in each tube (Eppendorf Safe-Lock Tubes, 1.0 mL, Eppendorf Quality™, colourless (Eppendorf, Hamburg, Germany)), and then immediately frozen. The samples were stored at a temperature of −70 °C until the testing time. Freshly defrozen portions were used for laboratory analyses. Before the analysis, the samples were defrozen at room temperature. At first, standard plates were prepared according to the Sun Red Biotechnology Company’s (Shanghai, China) instruction sheet. Biotin-marked antibodies, analysed material, and streptavidin were added. The volume of the analysed material and reagents depended on the assayed parameter. The plates were incubated for 60 min at 37 °C. Next, the plate was washed fivefold with a rinsing buffer. Chromogen A and B was added and incubated for 10 min at 37 °C. Inhibitor solution was added. Absorbance was measured with a wavelength of 450 nm, whereas Envision^®^ was applied in the analysis on the basis of a linear curve (EnVision Multilabel Plate Reader; PerkinElmer, Waltham, MA, USA). The methodology was performed in accordance with the manufacturer’s instructions and validated. Sensitivity range and linearity range for IL-1β, IL-6, IL-8 are presented in Table 2. The levels of interleukin 1 ß, interleukin 6, and interleukin 8 were provided as [pg/mL].

### 2.2. Statistical Analysis

Statistical analyses were conducted with the use of Statistica 13 (StatSoft, Inc. Tulsa, OK, USA). All data regarding continuous variables were presented as a ±standard deviation (±SD) and medians; qualitative variables are presented as number and percentages. Qualitative data were analysed with the chi-squared test or chi-squared test with Yates correction. The Mann–Whitney U test was used to compare continuous variables between the groups. The Wilcoxon test was applied to compare laboratory details before and after rehabilitation in the study and control groups. Differences at *p* < 0.05 were considered statistically significant.

## 3. Results

Table 3 demonstrates the analysis of differences between the study and the control group. No statistically significant differences between the groups were found in the evaluation of laboratory data. Statistically significant differences were shown in the second laboratory analysis IL-1β (*p* < 0.001), IL-6 (*p* = 0.040), and IL-8 (*p* = 0.044).

**Figure 2:**
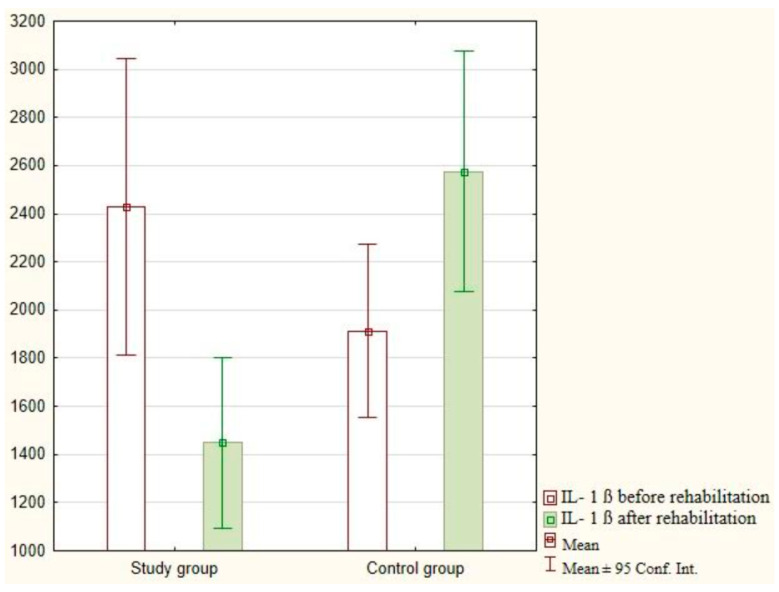
Analysis of IL-1 ß in the study and control groups.

The intragroup analysis conducted after the implementation of exercises with the use of virtual reality showed a decrease in the mean value of IL-1β (*p* < 0.001) in the study group and a significant increase in IL-1β (*p* < 0.001) in the control group. The research findings are presented in Figure 2.

**Figure 3:**
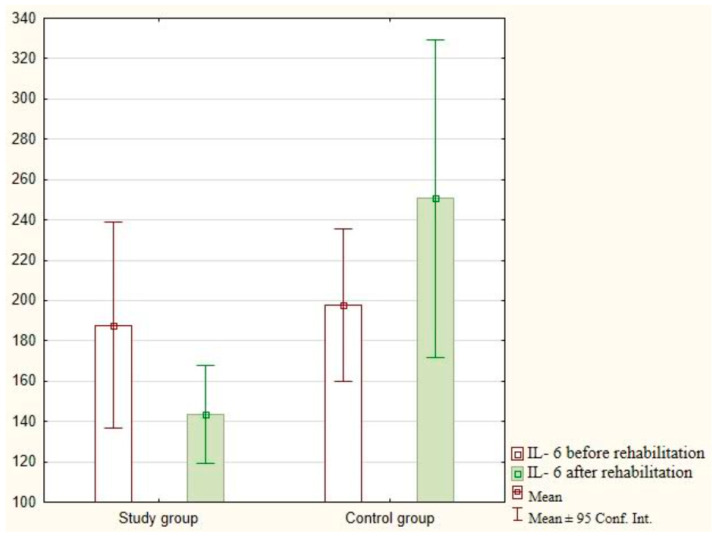
Analysis of IL-6 in the study and control groups.

The IL-6 analysis after the implementation of exercises with the use of virtual reality did not demonstrate statistically significant differences in the study group (*p* = 0.205) and the control group (*p* = 0.971). The research findings are presented in Figure 3.

**Figure 4:**
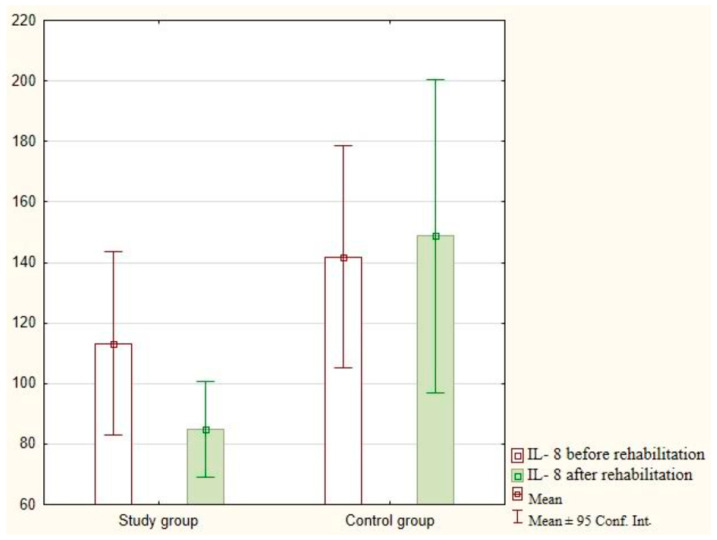
Analysis of IL-8 in the study and control groups.

The IL-8 analysis after the implementation of exercises with the use of virtual reality did not demonstrate statistically significant differences in the study group (*p* = 0.115) and the control group (*p* = 0.857). The research findings are presented in Figure 4.

## 4. Discussion

In the literature, there are reports of numerous benefits which derive from regular physical activity and its enhancement for the health and wellbeing of patients with chronic renal disease [10,27,28,29,30]. Despite the above, this group of patients features significantly lower physical activity levels than people with normal renal function [10,31]. An introduction of physical activity monitoring devices and innovative work out forms may enhance exercise attractiveness and become a motivation option for haemodialysed patients to work out regularly. An interesting idea may be to combine cycloergometer work outs with VR, as described in the literature [25,26,32].

The patients treated with renal-replacement therapy demonstrate increased synthesis and release of proinflammatory cytokines, such as IL-1β, IL-6, and IL-8 [33]. The rising number of publications concerning the effect of physical activity on the level of proinflammatory cytokines in the group of haemodialysed patients suggests that regular work outs may have a positive impact on the reduction in the levels of the same [34,35,36]. Nonetheless, this phenomenon remains unconfirmed in the scientific research.

This paper has been the first attempt to analyse the relationship between regular physical activity in virtual reality and the levels of plasma interleukin 1β, interleukin 6, and interleukin 8.

In the conducted analyses, the statistical analysis demonstrated a significant reduction in the level of IL-1β in the study group following regular exercises of low intensity with the application of virtual reality. In turn, in the control group which did not exercise, after 3 months, the level of IL-1β increased significantly. This may prove the thesis that regular work outs during haemodialysis decreased the level of the specified interleukin. Statistically significant differences were also shown in the second laboratory analysis of IL-1β. Lower values were reported in the study group.

Few researchers have evaluated the relationship between the level of IL-1β and physical activity or regular exercises in the group of patients included in the chronic haemodialysis treatment programme. Cruz et al. in their study also reported a reduction in the level of IL-1β following twelve weeks of exercising during haemodialysis [37]. On the other hand, in other papers, no statistically significant differences in the levels of IL-1β were found [38]. In the studies conducted by Tartibian et al., it was observed that regular low-intensity physical effort decreases the levels of IL-1β and IL-6 in a group of postmenopausal women [39]. The findings show that exercising systematically may significantly reduce the level of IL-1β irrespective of one’s age or health condition. According to Ding and Xu, aerobic exercises are the most effective method of working out, and low-to-moderate-intensity and mixed-intensity exercises are better when compared to high-intensity exercises in reducing IL-1β [40]. Regular physical activity has been shown to lower the level of interleukin 1β also in other diseases [41,42]. It should also be noted here that the available literature indicates that the level of IL-1β is decisively higher in patients with chronic renal disease than in the control group [43].

The level of IL-6 is oftentimes elevated in the group of chronic kidney disease patients, above all, in those treated with haemodialysis. The absence of or lowered physical activity may contribute to enhanced inflammation in CKD patients [44]. On the contrary, working out regularly is of special importance in chronic renal disease patients because it shows an anti-inflammatory effect [45].

The conducted study shows a significant difference in the level of interleukin 6 in the second laboratory analysis after exercising with the use of the NefroVR system. The research findings may prove the thesis that regular physical activity may reduce the level of plasma IL-6. Therefore, it would be worth expanding the study group and reanalysing the results.

Research concerning the correlation between physical activity and the level of IL-6 in haemodialysed patients is extensive. The studies by Gołębiewski et al., Moraes et al., and Dungey et al. did not show differences in the level of IL-6 after the recommended three-month bicycle work out during dialysis [38,46,47]. On the other hand, Cruz et al. and Liao et al. demonstrated a reduction in the level of IL-6 following regular aerobic exercises [37,48], and Meléndez-Oliva et al. showed a reduction in the IL-6 level in plasma after regular aerobic exercises and strength training [49].

Our research did not show statistically significant differences in the level of IL-8 in the studied patients after exercises with the use of the NefroVR system. However, a difference in the level of IL-8 was reported in the second laboratory analysis after the end of the 3-month training using VR. In the study group, the reported values were lower than in the control group. Sugawara et al. demonstrated reduced inflammatory cytokine levels, including those of IL-8, after low-intensity exercises [50]. Similar findings were reported by Cruz et al. [37]. Next, Dorneles et al. showed an increase in IL-8 post high-intensity exercises in individuals with obesity and regular body mass [51]. Moreover, a systemic increase in the level of IL-8 is observed in response to intensive exercise [52,53].

The data suggest that IL-8 is sensitive to exercise intensity. Other data show that systematic physical activity significantly reduces the level of proinflammatory cytokines, whereas the quantities of pro- and anti-inflammatory cytokines released under the influence of a one-time physical effort depend on its intensity and duration, as well as on the area of active muscles [54,55,56,57].

The levels of proinflammatory cytokines are closely related to M1 macrophage and macrophage polarisation in CKD. IL-1β and IL-6 are released by macrophages of the M1 phenotype. There are several studies which have investigated the macrophage polarisation during CKD [58,59,60].

Under physiological conditions, to inhibit excessive inflammatory responses, M1-type macrophages undergo transformation towards the M2 phenotype or undergo apoptosis. To further comprehend the relationship between higher levels of physical activity and improvement in the overall systemic inflammatory state, extensive studies are required, encompassing the measurement of numerous inflammatory factors [58,59,61].

### Limitation

This study has multiple limitations. The COVID-19 pandemics has limited the study to one dialysis centre and few participants.

In addition, the research results were affected by the worsening health condition of the CKD patients, renal replacement therapy, and concomitant diseases. It was challenging to find patients with chronic renal disease treated with haemodialysis willing to participate in this project to assess their physical activity. It was also necessary to choose a physical activity that the studied patients would be able to perform. The population of the study group during the research project declined for reasons beyond our control, such as renal transplant or death of project participants. The psychological aspect of introducing virtual reality could make the haemodialysed patients more willing to work out in the study. This could have been an independent factor with an impact on the study process. In the continuation of the study, it is necessary to increase the size of the study group as this would allow a more detailed analysis of the specified dependencies.

## 5. Conclusions

The conducted research demonstrated that regular exercise with the use of virtual reality might be related to a decrease in inflammation in the patients included in the chronic haemodialysis programme. It is key to encourage patients with end-stage renal disease treated with haemodialysis to exercise regularly because of the possibility of their proinflammatory parameters becoming reduced.

## Figures and Tables

**Table 1:** Group profile.

		Study Group (*n* = 39)		Control Group (*n* = 46)		*p*
Age, mean ± SD; Me		57.56 ± 17.61; 63.0		62.63 ± 15.47; 64.0		0.266 *
BMI, mean ± SD; Me		26.49 ± 3.76; 25.5		27.00 ± 4.69; 26.8		0.582
Sex, *n* (%)	Males	29	74.36%	29	63.04%	0.264
	Females	10	25.64%	17	36.96%	
Professional activity, *n* (%)	no	26	72.22%	40	88.89%	0.103^
	Yes	10	27.78%	5	11.11%	
Professional activity before the start of haemodialysis, *n* (%)	no	10	27.78%	21	46.67%	0.082
	Yes	26	72.22%	24	53.33%	
Type of job, *n* (%)	blue-collar	18	47.37%	18	54.55%	0.829
	white-collar	11	28.95%	7	21.21%	
	no job	9	23.68%	8	24.24%	
Currently smoking cigarettes, *n* (%)	No	30	76.92%	35	76.09%	0.868
	Yes	9	23.08%	11	23.91%	
Number of cigarettes per day, mean ± SD; Me		14.44 ± 6.13; 15.0		14.09 ± 7.41; 10.0		0.676 *
How many years ago quit smoking, mean ± SD; Me		9.71 ± 10.95; 5.0		16.67 ± 16.17; 13.0		0.520 *
Number of HD per week, mean ± SD; Me		2.95 ± 0.23; 3.0		2.98 ± 0.15; 3.0		0.805 *
Duration of dialysis [min], mean ± SD; Me		223.85 ± 20.47; 240.0		216.52 ± 28.92; 210.0		0.110 *
Concomitant diseases						
Diabetes, *n* (%)		5	14.71%	13	28.89%	0.224 ^
Arterial hypertension, *n* (%)		25	73.53%	32	71.11%	0.812
Epilepsy, *n* (%)		4	12%	3	7%	0.697
Ophthalmic, *n* (%)		8	24%	15	33%	0.484
Neurological, *n* (%)		2	6%	3	6.52%	0.745 ^
Treatment with another renal replacement therapy, *n* (%)		7	20.59%	8	17.78%	0.979

Legend: *n*—number of patients, SD—standard deviation, Me—median, HD—haemodialysis, BMI—body mass index, *p*—level of statistical significance. Notes: For data, test X^2^ was performed, *—Mann–Whitney U test was performed, ^—test X^2^ with Yates correction was used.

**Table 2:** Sensitivity range and linearity range for IL-1β, IL-6, and IL-8.

Interleukin	Sensitivity	Assay Range
1β	0.22 pg/mL	0.3–7500 pg/mL
6	1.0 pg/mL	1.5–300 pg/mL
8	0.9 ng/L	1–350 ng/L

**Table 3:** Analysis of laboratory data in the study and control groups.

	Study Group (*n* = 39)			Control Group (*n* = 46)			*p*
	Mean	Me	SD	Mean	Me	SD	
IL-1β—measurement 1	2428.99	1831.90	1794.83	1913.22	1348.35	1212.25	0.360
IL-1β—measurement 2	1448.34	1164.70	997.75	2575.40	1899.90	1658.73	<0.001 *
IL-6 measurement 1	187.83	143.46	146.86	197.53	150.44	127.66	0.353
IL-6 measurement 2	143.49	131.17	65.09	250.54	159.75	255.98	0.040 *
IL-8 measurement 1	113.28	104.94	75.33	141.83	98.23	119.40	0.476
IL-8 measurement 2	84.89	83.38	31.74	148.73	101.39	153.29	0.044 *

Legend: IL—interleukin, SD—standard deviation, Me—median, *p*—level of statistical significance, * statistical significance. Notes: measurement 1—before rehabilitation, measurement 2—after rehabilitation. For data, the Mann–Whitney U test was performed.

## Data Availability

The data that support the findings of this study are available from the corresponding author A.T.-S., upon reasonable request.
